# “You Probably Won’t Notice Any Symptoms”: Blood Pressure in Pregnancy—Discourses of Contested Expertise in an Era of Self-Care and Responsibilization

**DOI:** 10.1177/10497323211003067

**Published:** 2021-06-11

**Authors:** Lisa Hinton, Alison Chisholm, Beth Jakubowski, Sheila Greenfield, Katherine L Tucker, Richard J McManus, Louise Locock

**Affiliations:** 1 University of Cambridge, Cambridge, United Kingdom; 2 University of Oxford, Oxford, United Kingdom; 3 University of Birmingham, Birmingham, United Kingdom; 4 University of Aberdeen, Aberdeen, United Kingdom

**Keywords:** blood pressure, preeclampsia, self-monitoring, pregnancy, discourse analysis, information, responsibilization, qualitative methods, United Kingdom

## Abstract

Pregnancy is not a disease or illness, but requires clinical surveillance as life-threatening complications can develop. Preeclampsia, one such potentially serious complication, puts both mother and baby at risk. Self-monitoring blood pressure in the general population is well established, and its potential in pregnancy is currently being explored. In the context of self-monitoring, the information and guidance given to women regarding hypertension, and the literature they themselves seek out during pregnancy, are vital to perceptions of disease risk and subsequent responses to, and management of, any symptoms. Drawing on online, offline, official, and unofficial sources of information, discourses are examined to provide analysis of how self-responsibilization is reflected in contemporary information, advice, and guidance drawn from multiple sources. A paradox emerges between the paternalistic and lay discourses that seek to challenge and regain control. Findings are discussed in the context of Foucault’s governmentality and medical power.

## Introduction

Pregnancy is a unique phase in a woman’s life. It is a natural part of the life-course and accompanied by a social transition. It is not a disease or illness, but does require clinical monitoring and surveillance as complications can develop in pregnancy that can be life-threatening for mothers and/or their baby. Preeclampsia, a leading cause of maternal death and premature birth worldwide, is one such complication and puts both the mother and baby at serious risk (Shennan et al., 2017). The two diagnostic features of preeclampsia are hypertension (high blood pressure) and proteinuria (the leaking of protein into urine). Current U.K. antenatal care recommends that both of these are monitored routinely by health professionals throughout pregnancy, more frequently in those who are at higher risk (National Institute for Health and Care Excellence, 2008, 2019). Women are considered at moderate risk of preeclampsia in their first pregnancy, if they are overweight, above age 40, have previous or family history, or preexisting high blood pressure (National Institute for Health and Care Excellence, 2019).

Self-monitoring of blood pressure in the general population has become more common. Large trials have demonstrated its effectiveness in terms of blood pressure control (McManus et al., 2010). The potential for self-monitoring blood pressure in pregnancy to improve the detection and monitoring of hypertension in pregnancy is being explored (Tucker et al., 2017). Although Canadians include self-monitoring in their antenatal guidelines, and home blood pressure readings are the preferred method for out-of-office measurement for Canadian obstetricians, this is not the case widely (Tran et al., 2019). Up-to-date figures for how many women in the United Kingdom might be self-monitoring their blood pressure in pregnancy indicate that one in five pregnant women monitor their own blood pressure, and almost half of women with a diagnosis of hypertension self-monitor (Tucker et al., 2021). Offering further responsibility through self-management could be an obvious next step, as has been seen in the management of hypertension in the general population (McManus et al., 2018). In this context, the information and guidance given to women regarding hypertension, and the literature they themselves seek out during pregnancy, are vital to their perception of their risk status for the disease and their subsequent response and management of any symptoms. In this article, we use the theoretical lenses of responsibilization, medicalization, risk, and lay perspectives to explore these shifts. Although clinical monitoring in pregnancy targets a wide range of potential complications for the mother’s or her baby’s health, the focus in this article is on preeclampsia. It should be noted that there are wider implications beyond this single condition.

## Self-Care and Responsibilization

Policies that encourage patients to take greater responsibility for their health care are prevalent across the developed world (Armstrong, 2014; Vassilev et al., 2017). This shift, characterized as part of a neoliberal transfer of responsibility of care to the individual, entails the work of self-care, which can be both burdensome and empowering (Cohn, 2014; Department of Health, 2014; Henwood et al., 2011). The emerging literature on responsibilization provides important context here, as it explores these shifts in responsibility for personal health and individual agency (Lupton, 2016). Alongside these policies and social redistributions of power run the rapid development and expansion of digital technologies to support this self-care and expand the medical gaze (Lupton, 2016; Nafus, 2016). In 2020–2021, the social distancing requirements during the coronavirus pandemic rapidly escalated moves toward remote care, which have accelerated this trend (Royal College of Obstetricians and Gynaecologists, 2020). As Lupton has explored, digital health technologies can promote active “patienthood” performed through self-monitoring with the aim of taking control of one’s own health (Lupton, 2016). But these come with a cost and can be viewed as part of a politically driven project toward self-governance, which not only empowers individuals but also requires them to invest in new knowledge and skills (Vassilev et al., 2017). Petrakaki et al. (2018) explored the tensions between empowerment and self-discipline embodied in the discourses of technological self-care. Their study captured the perspectives of health policy makers, digital health experts, and patient organizations in the context of health policy that promotes the use of digital health technology to empower people to “take charge of their own health, by providing information, support and control” (Petrakaki et al., 2018, p.148). Examined were the perspectives of policy and technology developers on a landscape where patients are increasingly empowered as “co-producers” of their own health care. Also explored were the tensions between self-discipline and empowerment inscribed in the technologies and how they are “inextricably linked to a discourse of patient responsibilization” (Petrakaki et al., 2018, p. 151). However, their article did not capture the perspectives of clinicians or patients, and called for further research to unpack the different forms of agency that specific forms of digital technologies could afford.

This article adds to the literature on responsibilization through taking the specific clinical example of self-monitoring blood pressure in pregnancy and the challenges that emerge in the transition from an uncomplicated pregnancy to one requiring significant medicalization when blood pressure rises. Medicalization describes a process “by which nonmedical problems become defined and treated as medical problems” (Conrad, 1992, p.2). In uncomplicated pregnancy, medicalization has been considered to be inappropriate, as discussed below. However, in pregnancy, nonmedical problems can develop into medical ones. If a woman develops high blood pressure in her pregnancy, rapid medicalization—at least as far as her blood pressure goes—is important to preserve the health of the mother and her baby (Conti-Ramsden et al., 2019). Self-care in pregnancy to date has focused mainly on promoting healthy pregnancy and a healthy fetus, and not causing the fetus harm. The emphasis has been on good diet, appropriate weight, and reducing smoking and alcohol consumption. These can be seen as adopting lifestyle practices toward what a “responsible” pregnant woman does, but are not explicitly medicalized actions. Thus, self-monitoring of blood pressure represents women taking responsibility for their health care in new domains (Coxon, 2017; E. Martin, 2001).

## Resistance to Medicalization

There is a long history, in high-income settings at least, of resistance to overmedicalization and medical dominance of the female body (Boston Women’s Health Book Collective, 1976; Johanson et al., 2002; E. Martin, 2001) ranging from the impact of overmedicalized interventions to understanding how the media’s representation of the medicalization of birth has altered women’s perceptions of childbirth (Luce et al., 2016; S. Miller et al., 2016). It has been argued that the medicalization of birth has “gone too far,” leaving women as passive recipients in the childbirth process (Johanson et al., 2002, p. 892). One functionalist school of thought refers to the woman’s body as a machine (Wertz & Wertz, 1989) and the doctor as the mechanic or technician who fixes it (E. Martin, 2001). Henley-Einion (2003) describes birth as having become a “medical event” rather than a “social one.” A 2019 study examining the experiences of health care professionals and the medicalization of childbirth found “most midwives associate medicalization with the general perception that a condition defined as pathological needs surveillance and intervention” (Prosen & Krajnc, 2019, e176). Monitoring and testing takes this further. In her 2015 review of the social processes of reproduction, Almeling described self-monitoring and testing as placing women in “the role of ‘moral pioneers’ who must decide whether to learn this information and how to respond to it” (Almeling, 2015, p.426). Lupton argued that in the interests of promoting the health and well-being of their fetuses, pregnant women are subject to imperatives, which expect them to engage in regimes of self-regulation and discipline, and they become the focal point for regulation, monitoring, and control (Lupton, 2012). She traced discourses of fetal risk and notions that the pregnant woman is expected to “exert her ethical responsibility” (Lupton, 2012, p. 337). She is encouraged to regulate and monitor her activities, to avoid sources of harm (alcohol, tobacco, caffeine, and various foods), consume enough water and vitamins, and avoid strenuous lifting. She is called on “to ‘do your research’ by reading pregnancy books to ensure they are informed, participate in prenatal screening and testing for fetal abnormalities, exercise regularly, etc.” (Lupton, 2012, p. 329).

## Pregnancy and Childbirth Within the Risk Society

There are also broader discourses of risk in pregnancy and childbirth, where surveillance is put in place to mitigate risk. Despite the fact that obstetrics is considered a high-risk and high-litigation area, accounting, for example, for 60% of all the United Kingdom’s National Health Service’s litigation claims, there is relatively little scholarly work on childbirth as part of the risk society (Scamell, 2014; Yau et al., 2020). Scammell argued that thinking in terms of risk is a process of mitigating the unknowns of childbirth, “minimizing the unpredictability of the future in an attempt to improve the outcome” (Scamell, 2014, p. 921). Citing Giddens, she suggested that risk implies activities of security, such as monitoring of pregnant women for signs of disease (Giddens, 1991). The monitoring that women surrender to during pregnancy can be seen as activities of security by the clinical community and health care system. These are intended to manage this risk, to avoid poor outcomes for the mother and/or baby. But they come at a cost to women. Evans and O’Brien’s (2005) research on gestational diabetes revealed the contradictory meanings of monitoring for pregnant women and their health care providers. Their article called for a richer understanding of what it means to women to be in control during an at-risk pregnancy, and of the salience of conversing with others for information and support. “To sustain a medicalized view of pregnancy, particularly one deemed at risk, can distort the women’s lived experience of her pregnancy and jeopardize her sense of personhood and normalcy” (Evans & O’Brien, 2005, p. 79). As technology progresses, doctors and midwives face increasing pressure to intervene to nullify any perceived threat of a poor outcome for the mother or the baby; invoking a public belief that all maternal and neonatal deaths could be prevented or avoided (Johanson et al., 2002). Women likewise feel a pressure to do the right thing to achieve a successful pregnancy and avoid poor outcomes (Coxon, 2017). These dynamics are further complicated by women in developed *and* developing countries becoming at higher risk in their pregnancies due to increased maternal age, obesity, and associated health conditions. Our article extends previous scholarship on the shift beyond domains where women have historically been expected to exercise self-governance, such as smoking, weight, and drinking alcohol, into new domains where they are being co-opted to share in what have heretofore been clinical surveillance responsibilities (Hammer & Inglin, 2014; Wagnild & Pollard, 2020).

## Lay Expertise

An emerging discourse in public health is the rising prominence of lay expertise (Popay, 2018). Originating from Gramsci’s theory of organic intellectuals, which theorized that every social group in the economic, social, and political fields creates a “strata of intellectuals which give it homogeneity,” we now have rich scholarship on lay knowledge and expertise (Gramsci, 1992). This theory gained prominence after the 1980s’ AIDS outbreak. Epstein credits the gay and people living with HIV/AIDS (PLWHA) community for driving the reform of clinical research as they became genuine and valuable participants in the construction of scientific knowledge about the disease (Epstein, 1995). Patient activism and lay expertise became more commonplace, and similar movements subsequently emerged in women’s health. The Boston Women’s Collective and Our Bodies Our Selves in the United States (https://www.ourbodiesourselves.org) were mirrored by the natural birth movement with campaigners such as Sheila Kitzinger and Janet Balaskas, and the emergence of the National Childbirth Trust in the United Kingdom. Although the pregnancy community has not needed to amass credibility and scientific language in the same extensive manner as the PLWHA community, the availability of pregnancy-related information in the digital age has created a level of lay expertise within this group (Johnson, 2014; Lowe et al., 2009; Mackintosh et al., 2020; Song et al., 2012). A contributing factor is the increased use of pregnancy apps; pregnant woman are using apps in greater numbers, whether to track their pregnancy progress or for pregnancy information (Kraschnewski et al., 2014; Thomas & Lupton, 2016). The rise of the internet has also led to women seeking information online from sources that are not grounded in medical expertise, but based on anecdotal evidence or lived experiences.

## Information Seeking

In the context of these contested debates around medicalization, risk, and knowledge, maternity care is highly likely to be influenced by where women gather information and whether or not they trust that information. Pregnancy is a particularly important time for women, and information seeking as they embark on a journey into the unknown, whether it is their first or a subsequent pregnancy, is particularly salient. We know they garner much of their information from friends and family, TV programs, the written information distributed by blogs, pregnancy books, websites, online forums, and apps, as well as from health care provider leaflets and websites (Hinton et al., 2018; Luce et al., 2016; Prescott & Mackie, 2017; Roberts et al., 2017).

Understanding more about what is said and what is not said is crucial as we seek to support women in healthy pregnancies. Blood pressure monitoring, although often experienced by women as a routine and unworrisome part of routine care and normal life (Hinton et al., 2017), can rapidly be reframed as a key component of a medicalized pregnancy if blood pressure rises to concerning levels. In this article, we analyze the discourses at work in the information women are provided about blood pressure in pregnancy and the information they seek out. In doing so, we add to contemporary scholarship by providing a worked example of an environment for self-responsibilization and the social construction of risk in the potentially highly charged clinical and personal setting of contemporary pregnancy. These social processes were accelerated in 2020 by the rapid move to remote antenatal care in response to the coronavirus pandemic in many settings. Information provided to, and sought out by, women needs to be understood not only clinically—what information they are given and seeking—but also socially. The research aims included exploring the information provided formally and informally to women about hypertension during pregnancy, and the information women themselves share online about their pregnancy. In drawing on both online and offline sources of information, the article provides a new analysis, informed by theoretical concepts of risk, self-responsibility, medicalization of pregnancy, and lay expertise, that reflects contemporary cherry-picking of information, advice, and guidance from multiple sources.

## Method

### Discourse Analysis

A discourse analytic approach was adopted. Discourse can be conceptualized as “*the ways that a topic is spoken of*” (Carabine, 2001) and discourse analysis as the close study of language in use (Wetherell et al., 2001). It is based on the premise that language is not simply a vehicle for transmitting meaning encoded by the sender and decoded by the receiver. Rather, language is “constitutive,” a site where meanings are created and changed, and is situated within particular social and cultural contexts. Through language, value is attributed or denied, people are categorized together or separated as different. By considering its situated use, it is possible to understand what is *being done with* language.

Shaw and Bailey distinguished three levels at which discourse analysis can be carried out: the microlevel, where attention is paid to the language itself, its vocabulary, structure, and function; the macrolevel, at which language and ideology in society are studied; and an intermediate, mesolevel, where the focus might be the connections between language and its broader social and cultural contexts. The analysis in this study is situated at a mesolevel, with a starting point that “discourse guides certain ways of talking about a topic, defining ‘acceptable’ ways to talk, write or conduct oneself and that this can serve a range of social functions” (Shaw & Bailey, 2009, p. 415). Carabine argued that discourses are productive in two ways: They construct a particular version of a topic as real and they have power outcomes (Carabine, 2001). In applying discourse analysis to the ways that self-monitoring blood pressure is spoken of, this article identifies how meanings are constructed about blood pressure, how and by whom it is monitored in pregnancy, and the outcomes of these discourses in terms of power.

A Foucauldian approach to discourse analysis examines relationships between discourse, power, and knowledge (Downing, 2008). Carabine wrote that “discourses are historically variable ways of specifying knowledges and truths, whereby knowledges are socially constructed and produced by effects of power and spoken of in terms of ‘truths’” (Carabine, 2001, pp. 273–274), and argued that discourses are powerful because they specify what is and what is not, and that some have more authority and validity than others. A discourse contains subjects, supports institutions, and reproduces power relations (Shaw & Greenhalgh, 2008).

### Data Collection

A wide range of information was sought about self-monitoring blood pressure in pregnancy, focusing on material that is available to women during pregnancy without searching for information specifically about preeclampsia. Sources included information given directly to pregnant women by health care professionals in the United Kingdom (locally produced materials came from the geographical areas in which the patient and public involvement [PPI] group and the health care professionals who commented on the searches were based), information women find themselves published online and offline, and discussion generated by women in online fora, on social media, vlogs, blogs, and so forth. The search was guided by the inclusion and exclusion criteria listed and carried out in January and February 2017.

Inclusion criteria are as follows:

- hold information related to pregnancy in general or in health during pregnancy and
- in the public domain

Exclusion criteria are as follows:

- Non-English language sources;
- Nonwomen focus (e.g., pregnancy for fathers);
- Focus on postbirth, not pregnancy;
- Did not offer information (e.g., journal/diary for parent to complete, baby names);
- Narrow focus (e.g., apps for timing contractions, monitoring the baby’s movement); and
- Password-protected sources or those that required membership to view.

The following categories of data were identified:

1. Formal/official materials routinely handed to pregnant women by health care professionals,
2. Professionally produced websites,
3. Email alerts,
4. Books,
5. Magazines,
6. Apps (mobile software applications designed for smartphones),
7. Discussion forums (user-generated health-related content),
8. Social media,
9. Blogs/vlogs,
10. YouTube videos, and
11. Podcasts.

The categories were used to structure the search for information. The searches were designed to approximate “real world” approaches to searching to identify content that women are most likely to find. Categories were developed by the research team and a PPI group convened to support and inform the search. The women were invited to take part by a research midwife known to the research team. The midwife was asked to select women who varied in terms of their parity and their risk factors for pregnancy hypertension and preeclampsia. The PPI group was made up of four women, including two who were pregnant with their first baby and two pregnant with subsequent babies, three of whom had no risk factors for preeclampsia and one with risk factors. No formal consent process was required, but women in the group were assured that their contributions would be reported anonymously and they would not be identifiable in any publications. They were asked about the methods and approaches they used to find information about blood pressure, and were asked to identify any sources of information that were missing from our search results. Additional sources identified by the PPI group were added.

In some categories (e.g., information routinely provided by health care professionals), sources of information were identified based on the experience and knowledge of members of the research team and the PPI group. In others (e.g., books), the most accessible, popular, and well-used information was identified using online searches of popular websites. All online searches were carried out using incognito browsing. These were supplemented with visits to a public library and discussion with the PPI group. Midwives, primary care doctors (general practitioners [GPs]), and obstetricians known to the research team through professional contacts were asked to comment on the list and identify any missing sources. As with the PPI group, no formal consent process was required, but they were assured that steps would be taken to ensure their contributions were anonymous and they would not be identifiable in any publications. Search methods for each category, along with search terms where relevant, and results can be found in Supplemental Table S1.

### Research Ethics

Our university ethics board (Central University Research Ethics Committee, Oxford University) was consulted and confirmed that the study did not require ethical review, although we acknowledge ethical approval may have been required in other countries or by other universities. Only posts in the public domain were used and anonymity has been protected by not using the posters’ given pseudonyms and ensuring as far as possible that no details were included from posts that could render them identifiable. The National Institute for Health Research confirms that ethical approval is not required for PPI activity.

### Analysis

Throughout, the analysis was an ongoing process of refining the research focus to pinpoint the discourses to explore and to address issues of power, control, relationships, knowledge, expertise, responsibility, meanings, and outcomes. The discourse analytic methods adopted in this study draw on Carabine’s (2001) approach to Foucauldian genealogical discourse analysis, looking in particular for the mechanisms of power at work in these texts and offering a description of their functioning. Analysis began with the selection of a topic and sources of data (as described above). The next stage was familiarization with the data by reading and rereading the text. NVivo 12 was used to organize and analyze the data. Hinton, Chisholm, and Jakubowski grouped the data into formal and informal sources of information to better understand the differences and discourses between these groups. Formal data were assessed as medical literature, both online and in print, and information provided by an official organization or medical professional. Informal data were assessed as information provided by nonmedical personnel and were broadly online forums and personal blogs. This was followed by a process of identifying the themes in the data, which authors Hinton, Chisholm, and Jakubowski carried out independently by searching the texts for categories and objects of discourse. The data sources were divided between authors, who each independently identified codes and subsequently met to discuss and compare themes, leading to agreement on a set of common themes to be applied to the full data set.

The next stages of the analytic process were to look for evidence of interrelationship between discourses and to identify discursive strategies and techniques that are employed (how discourses were deployed, how they are given meaning and force). Questions that guided this process were as follows: “Are women and health care professionals presented in a positive, neutral or negative way?” “Who is identified as being responsible for high blood pressure and monitoring/detecting it?” “What language and tone are used to refer to blood pressure and self-monitoring? Is it judgmental or moralizing?” and “How is risk presented?” The researchers particularly reflected on the ways that information is presented and how this influenced the women’s relationships with their antenatal care providers (doctors and midwives) and their own sense of personal responsibility for their pregnancy. During this stage, we remained alert to silences, resistances, and counterdiscourses. These silences and resistances were most apparent in the way various sources indicated weak relationships between women and health care professionals, reflected in the way that women were sharing knowledge and lay expertise online instead of engaging with their health care professionals, and in the professional literature that limited the role of pregnant women in their own care. The final stage of the analytic process was to identify the effects, and potential effects, of the discourses and the implications of these for formulating policy.

## Results

### Reflections on These Data

Discourses are historically situated and should be considered in relation to time. Some of the sources included in the analysis were posted several years earlier (the earliest being 2007) but these posts are still available and could be found in a present day online search (late 2020). The wide variety of texts identified reflect the rapid increase of health information and guidance that women are presented with in contemporary antenatal care. It was not possible or practical to capture everything or assess how many of these sources an individual woman may access. Indeed, she herself may not be able to track back all the sources of her knowledge acquisition. We no longer live in a world where the book on the shelf, or the leaflet on the table, are the only places we might have read something. But this patchwork approach to knowledge building is how we live today, an approach with parallels to the concept of bricolage (using whatever is at hand to create something new) as discussed by Lévi-Strauss (1962) in *The Savage Mind*. The discourses at work within this landscape have profound impacts on contemporary pregnancy.

The search revealed that the categories are not distinct and mutually exclusive. For example, *Emma’s Diary* (a publication with information and advice about pregnancy made available in the United Kingdom by family doctors [GPs] and midwives to pregnant women) falls into four categories (see the supplemental table): It is routinely handed to women by midwives (category 1 in the list above) in magazine-style format (category 5), its content is available on a website (category 2), and women can register for email alerts (category 3). In the table of search results, information sources that fit in more than one category appear under each category in the table. The findings revealed two broad discourses at odds with each other: a paternalistic discourse, where responsibility and knowledge rest with doctors and midwives, and the lay perspectives and voices. These two discourses are described and the consequences of the tension between them explored.

### “Relax,” “Be Reassured”: Paternalistic Discourse and Its Consequences

In the formal information women were given or that they accessed, the overriding discourse was of benign Foucauldian surveillance, a scaffold of monitoring that was there to protect mother and baby. Preeclampsia and its risks and symptoms were explained, largely in sections dedicated to the later stages of pregnancy or complications, although routine checks were described throughout. Books and websites laid out the antenatal care pathways, with the regular checks and monitoring that were administered and interpreted by clinicians. Women were reassured that there was a well-defined and structured care pathway that would identify an emerging problem, with clinician and patient each with their predefined roles and expectations. Although the warnings were dire, women were reassured. “Fortunately in women who are receiving regular medical care, pre-eclampsia is almost invariably caught early on and managed successfully” (Murkoff, 2016, p.547).

Doctors and midwives were presented as “in charge.” The power, knowledge, and the responsibility rest with them. All the woman had to do was show up to her appointments. Signs of preeclampsia would be monitored through blood and urine tests that clinicians would administer. Although this may be because, at least until recently, the technology to measure blood pressure has been inaccessible or unaffordable to individual women, the paternalistic language still dominated.

your midwife will look for any signs and symptoms of the condition at your antenatal checkups. (Emma’s Diary)

We worry these could be signs of pre-eclampsia. (Fogle, 2016)

Test results were interpreted and risks assessed on the basis of clinicians’ expertise and knowledge. Women were repeatedly reassured that the responsibility for monitoring their ongoing health lay with the midwives and doctors; it was they who would be looking for clues for the disease.

That’s why your midwife or doctor will carry out tests for these signs at every antenatal appointment throughout your pregnancy, just to be on the safe side. (Babycentre, no date)

At your booking visit you will have been asked a number of questions by your midwife or doctor. From this information your doctor will have identified whether you are at risk. (Hospital patient leaflet, NHS)

The consequences of these discourses are that the women were cast as passive recipients of care and kept safe by clinical surveillance. They had prescribed roles, which were primarily to engage with the pathway actively, turn up to their appointments, and report any worrying symptoms. But they had little agency or responsibility (Armstrong, 2014). The stealthy nature of preeclampsia—a condition that is often symptomless, or masked by other physical changes that are normalized in pregnancy (swollen ankles, fatigue) until quite severe disease has developed—reinforced this passivity.

so, it is very common and normal for your fingers and legs to become slightly swollen. Having said that, if you notice that your face, fingers or legs have suddenly become much puffier or swollen these may be the early signs of pre-eclampsia. (Regan, 2005, p. 207)

The reassuring tone on the National Health Service (NHS) website—“you probably won’t notice any symptoms of either of these but your GP or midwife should pick them up during your routine antenatal appointment”—was softened elsewhere (NHS Choices). But the messaging was unclear. Although overall responsibility lay with the health professional, women are required to be vigilant and report symptoms, even though they were told symptoms can be masked.

Because the symptoms of pre-eclampsia aren’t usually noticeable to mums-to-be themselves it’s crucial that you keep all your antenatal appointments where you’ll be checked for signs at each visit. (Bounty)

Relax. Worrying about your blood pressure will only send the reading higher. Besides a slight increase at one check-up is probably nothing to worry about. (Murkoff, 2016, p. 204)

A “slight increase” was not defined, so information was not provided for women to understand its significance. Although authors might have been trying to keep the tone light, seeking to minimize worry and reassure, it could also be interpreted as disempowering. It might also be wrong, as high blood pressure readings on one occasion are regularly acted on, especially if particularly high (National Institute for Health and Care Excellence, 2019).

## “Any Advice?” “I Don’t Really Know What I’m Talking About But I Just Wanted to Offer Some Reassurance.” Medical Expertise Contested by Lay Knowledge/Discourses Seeking Control

By contrast, the discourses at work in the lay data were of worry, advice seeking, lack of confidence, a challenge to medical authority, and, crucially, seeking control. Pregnant women are not alone in going online to seek information and reassurance. This space revealed a blend of semiofficial looking advice and guidance and lay advice seeking and knowledge sharing.

There were often small fragments of conversations between women seeking temporal advice in a moment of anxiety or worry, albeit available years later. Women seemed to be looking for reassurance—“Should I go to the midwife?” “Any advice?” “I’m panicking like mad,” “I just feel really confused” (Mumsnet, 2013). Whereas official sources and advice books will have been written in a measured, thoughtful, and largely evidence-based way, these online conversations were informal, chatty snippets, written in haste, in the margins of daily life. As one forum comment stated, “Above should have read ‘quick message as between tube [train] stations’!” (National Childbirth Trust, 2016) These could be dismissed as partial, biased, or anecdotal and skewed toward the extreme or more frightening examples. The postings tended to be skewed toward questions, fears, and problems for which women were seeking solutions. Rather like TripAdvisor hotel and restaurant ratings, there was a bias toward brilliant or terrible experiences (Fong et al., 2017), so presentations of positive birth experiences did not come to the fore. But the fact that these postings were easily found in online searches, are still available, and come up alongside more formal sources means that they warrant study and consideration. We know that women build their knowledge from multiple sources (Hinton et al., 2018; McLeish et al., 2020; Sunstein, 2001), so what is happening in these spaces informs the whole. There was a variety of tone. The voices often “spoke” with authority and although a few acknowledge their lack of clinical knowledge, their immediacy could be powerful.

Good luck, I don’t really know what I’m talking about but just wanted to offer some reassurance. (Mumsnet, 2010)

### Control

There was a discourse of seeking control and empowerment and in some way challenging the surveillance in place to pick up preeclampsia. Women were worried about having to take medicines and being hospitalized, and frequently shared tips to try and ward these off. Some were more evidence based than others (rest, relax, cut down on salt, eat protein.)

So, the only thing I would advise is if you feel faint lie on your side rather than your back. I don’t know if this is scientifically accurate but I would have thought that blood reaches the baby more easily than it does the brain as the baby is below the heart. (Mumsnet, 2010)

There was also a discourse that challenged the authority of clinicians and their right to make decisions for the women, “wow how to make a mountain out of a molehill!!!!” (Babycentre Community, 2014). Rather than acknowledging the expertise and motivations of health care staff, they viewed their concern and monitoring as something to fight against.

They tried this on with me for my first and tried to tell me I had PE—I went for monitoring every two days and proved I didn’t have PE but they still wrote it in my notes. I still went ahead. (Babycentre Community, 2014)

If I’m being honest I think you’ve probably been unnecessarily scared by the docs here wrt the PE. (Mumsnet, 2008)

In a Mumsnet thread, the language used was also authoritative; “first of all it is not normal for blood pressure to be raised during pregnancy; if anything, it should be at a lower level than when not pregnant,” and encouraged women to challenge and take control through seeking alternative medical advice, “If I were you I’d get a second opinion” (Mumsnet, 2007).

Have checked out the links & taken heart by the stories and sensible advice here. Thank you so much—really helps to know I’m not the only one and that there can be good, non-interventionalist outcomes in hospital and equally that it is possible to have a safe home birth if its carefully monitored. Feel a bit more in control now—it was just such a horrid shock at 36 weeks—wasn’t expecting to have my plans changed! (Mumsnet, 2007)

Intertwined was a language of advocacy and agency. Despite the architecture of a care pathway designed to minimize the disease (and with success, as evidenced by recent Confidential Enquiries into Maternal Death and Morbidity [Conti-Ramsden et al., 2019]), women talk a language of self-advocacy and challenge. Relationships with health care professionals were complicated. On one hand, women contested their authority, and yet on the other hand, feared bothering staff with their concerns. Risk and risk acceptance came through strongly in these extracts.

Right I’m going to speak frankly—you are being foolish now. As I read it your history is:

Previous high BP in pregnancy Protein in recent urine visual disturbance headache One high reading, one low reading. There are some major red flags here and you need to stop worrying about what people will think and get checked tonight. Ring the labour ward for advice or go to A&E. You are potentially risking your baby and your life if you ignore this collection of events. (Mumsnet, 2011)

## Whose Responsibility?

Thus, where the responsibility lies, for self-care, for surveillance, for medical action, became blurred. Although the official sources made it clear that the responsibility lies with the clinicians, embedded in the antenatal care pathway, a close analysis of this online space not only seeks to challenge and undermine this (as seen above) but also taps into discourses of personal responsibility. Advice was sought and given, top tips shared. There were a few voices that cautioned privileging this lay knowledge over the expert and women expressing a choice for pat/maternalism:

Don’t mess about doing own blood pressure, leave it to experts. Good luck x. (Mumsnet, 2011)

Electronic bp machines are not accurate in maternity cases! Any midwife would tell u not to use it!! Blood pressure fluctuates all the time in pregnancy! Let the professionals do their job. (Mumsnet, 2013)

## Discussion

Many women are motivated to take responsibility for themselves in pregnancy, legitimately seeing problems with overmedicalization. However, once a potentially dangerous medical problem is superimposed onto that pregnancy—here high blood pressure—a stark mismatch emerged between the paternalistic discourse of “relax and be reassured” and medical expertise being contested by lay knowledge and discourses that seek to regain control. This contest has been so since birth was medicalized. These findings speak to the dual discourses at work in pregnancy that have inherent tensions within them. There is a discourse in which pregnancy is set up as normal and healthy. Women are encouraged to carry their notes, choose their care, and retain control. But alongside this is a counternarrative of surveillance and discourse around the labor of being a responsible mother and managing risk. The shock that accompanies a diagnosis of preeclampsia comes from being tipped from one discourse to the other, into abnormality and potential catastrophe, where expectations of a happy and healthy pregnancy are not met. These discourses sit in interesting opposition to the authority of the official discourses. Where does responsibility for care and decision making lie? This article presents two novel findings. One is the mobilization of online resources to resist this surveillance and control. The other is the extent to which, in this clinical domain, health professionals seek to retain control, which is at odds with the push for self-management elsewhere in health care.

## Other Literature

Pregnancy guides and manuals are not new, as chronicled in Seigal’s history and rhetorical analysis, “The Rhetoric of Pregnancy” (Seigal, 2014), which linked Foucault’s work on language, discourse, and power (Foucault, 1972), and Oakley’s earlier work on the medical care of pregnant women (Oakley, 1984). But the range and number of sources of information and support in pregnancy that women draw on are proliferating (Hinton et al., 2018; Mackintosh et al., 2020), with digital technology playing an increasingly dominant role. It is anticipated that the use of self-monitoring devices (such blood pressure monitors or Doppler machines to hear the baby’s heart beat), apps (such as ovulation trackers), and other online sources will influence maternity care and should be considered in the future planning of health care provision, suggesting a reconfiguration of the responsibilities of health care providers and pregnant women. The shift to self-care/monitoring is a central piece of this shifting landscape and one that has accelerated with the 2020–2021 coronavirus pandemic with an abrupt reduction in face-to-face care. It seems likely that once the immediate crisis has passed, some of these changes will endure.

There is an emerging body of literature that seeks to understand the context and sociocultural impact of digital technologies on contemporary pregnancy, with pregnancy apps at the newest frontier of information provision, and commercialization (Johnson, 2014; Thomas & Lupton, 2016). In their analysis, Thomas and Lupton identified two distinct forms of pregnancy apps, based around “threats” and “thrills” and argued that “taken together, the apps rest on neoliberal ideologies concerning the management and responsibilization of the self/body” (Thomas & Lupton, 2016, p. 506). Pregnancy becomes a space for entertainment as well as a period of danger. Conversely Johnson’s study of social media and the self-management of pregnancy and early motherhood framed apps as empowering technologies that enable women to take control of their experiences and “more efficiently enact their expert patient role” (Johnson, 2014, p. 346). In their recent study of women’s use of online resources and apps in the perinatal period, Mackintosh et al. (2020) concluded that online information retrieval and digital self-monitoring are increasingly integral to women’s self-care during pregnancy and offer opportunities to support escalation of care and shared decision making.

These rapid and contemporary digital developments, often unregulated, are taking place alongside the broader neoliberal shifts discussed by Petrakaki and colleagues (2018). Their work links with the broader literatures of responsibility and agency (Armstrong, 2014), reproductive citizenship (Lupton, 2012), pregnancy and surveillance (Greene et al., 2017), and childbirth within a risk society (Boardman, 2017; Coxon, 2014; Scamell, 2014). The findings we present here highlight the ongoing tensions between power/surveillance and self-responsibility. Giddens (1991) drew attention to the positive power of surveillance that “attempts to tackle uncertainty and ward off danger,” which conflicts with agency and self-responsibility discussed by Petrakaki where digital health technologies are seen as part of an enterprising identity for patients that service economic, neoliberal needs. In a recent editorial, Coxon explored current theorizing of risk in pregnancy and birth and argued that although prominent theorists of sociocultural understandings of risk, such as Giddens and Beck, presented the concept as resting on individualization through freedom from the traditional structures of late modern society, this does not hold (Coxon, 2017; Petrakaki et al., 2018). Hallgrimsdottir and Benner’s work explored the moral responsibilities of pregnant women through “maternal hygiene manuals” published at the turn of the 20th century, suggesting that self-governance in mitigating risk in pregnancy has been around for longer than we might think, and that it endures (Hallgrimsdottir & Benner, 2014). Miller observed that traditional structures continue to affect women’s experiences of motherhood: “their expectations will be shaped by and through expert systems of authoritative knowledge as they negotiate the ‘risky’ and morally underpinned path to ‘responsible’ motherhood” (T. Miller, 2005, pp. 48-49). The findings presented here add to this narrative, but the shifts toward the increasing reliance on online/digital resources and provision of remote care will have consequences for how women will navigate the competing discourses identified in our analysis, as well as for their relationships with, and expectations of, the clinicians who provide their care.

Although policy makers envisage a revolution in public health, powered by the rapid development of digital technology and new ways to share knowledge online, it is unclear how these shifts in power and knowledge will play out in highly paternalistic sectors of health care. This links to the concept of governmentality, work in the late 1990s that explored the decentered nature of power in contemporary societies. Martin and Waring suggested that Foucault’s nascent concept of pastoral power can be helpful in uncovering and explaining some of the complexities of how these discourses intertwine. They argue that although many examinations of governmentality portray a coordinated, or even monolithic, governmental discourse, this no longer has relevance. “It is difficult to argue that late modernity is characterized by a single unified regime of truth; indeed Foucault (2008) himself argued for the coexistence of multiple truth claim” (G. P. Martin & Waring, 2018, p.1305). The multiple sources of information explored in this study have parallels with these multiple truth claims and raise issues for the increasingly decentered nature of medical power in contemporary societies. They also have relevance for the plural preferences of pregnant women, some of who may wish to resist medical power, but others who may reject neoliberal responsibility.

## Strengths and Limitations

A key strength of this article is that it brings together an analysis of online and offline materials about blood pressure and blood pressure monitoring in pregnancy that women access. Although prior work has examined the new digital frontiers, this analysis seeks to provide a more real-world reflection of what women access and consume by way of information and advice in contemporary society. However, only a partial snapshot is offered, bounded by time and a high-income, northern hemisphere location, and through the lens of written materials. This research was undertaken in the United Kingdom, but the international nature of many of the data accessed suggests these tensions may manifest in other settings. We are aware that some online posts are now quite old, but they are still available at the time of publication and reflect that online discussions about these issues have been going on for a long time now. With these data, we are unable to offer a more intersectional approach to this topic, which warrants more research. This article does not hear directly from women or health care professionals. The focus of this article has been on just one aspect of pregnancy, the management of blood pressure, but we suggest that our findings have wider relevance to the interrelationships between women during their pregnancies, their health care providers, and the information they are provided with or seek out.

## Conclusion

Why does any of this matter? Women need clear information and trusting relationships to ensure good care during their pregnancy. This is particularly the case when illness is superimposed. This article has argued that these paradoxes could become increasingly stark as we move toward increasing personal responsibility and self-management in pregnancy, which may well be accelerated by the development of cheaper and more readily available monitoring equipment as well as disruptions in care as seen during the 2020–2021 coronavirus pandemic. These findings illuminate that the neoliberal push for self-management—supported by the web—appears, for now, to come up hard against professional paternalism in this contested discursive space. Is this tension resolvable, and who wins? Our analysis indicates that, at present, women are required to manage these tensions on a case-by-case basis. But this is a fast-changing landscape. Clinical studies are underway to build the evidence base for self-monitoring blood pressure during pregnancy, and the coronavirus pandemic has accelerated the implementation of remote care in some settings. The landscape in which these relationships are cast is changing rapidly. As the evidence for the use of self-monitoring of blood pressure in pregnancy emerges, a renegotiation of these dynamics may well be required from both sides.

## Supplemental Material

sj-docx-1-qhr-10.1177_10497323211003067 – Supplemental material for “You Probably Won’t Notice Any Symptoms”: Blood Pressure in Pregnancy—Discourses of Contested Expertise in an Era of Self-Care and Responsibilization Click here for additional data file. Supplemental material, sj-docx-1-qhr-10.1177_10497323211003067 for “You Probably Won’t Notice Any Symptoms”: Blood Pressure in Pregnancy—Discourses of Contested Expertise in an Era of Self-Care and Responsibilization by Lisa Hinton, Alison Chisholm, Beth Jakubowski, Sheila Greenfield, Katherine L Tucker, Richard J McManus and Louise Locock in Qualitative Health Research
