# Supplementary material for: “You Probably Won’t Notice Any Symptoms”: Blood Pressure in Pregnancy—Discourses of Contested Expertise in an Era of Self-Care and Responsibilization
Source: Qual Health Res. 2021 Jun 11;31(9):1632–44. doi: 10.1177/10497323211003067 (PMC8438769; doi:10.1177/10497323211003067)
Supplement: sj-docx-1-qhr-10.1177_10497323211003067 – Supplemental material for “You Probably Won’t Notice Any Symptoms”: Blood Pressure in Pregnancy—Discourses of Contested Expertise in an Era of Self-Care and Responsibilization [file sj-docx-1-qhr-10.1177_10497323211003067.docx]

**Table 1**

| **Category of data** | **How selected** | **Results** |
| --- | --- | --- |
| Formal/official materials routinely handed to pregnant women by healthcare professionals | Researchers, PPI group, HCPs (including midwives, GPs and obstetricians) | **OFFLINE**  Maternity packs & patient information pages and leaflets  Department of Health and Social Care Guide to pregnancy  **ONLINE**  Emma’s diary  NICE  Bounty |
| Professionally produced websites (govt. bodies, charities, health organisations, commercial organisations) | Researchers, PPI group, HCPs (including midwives, GPs and obstetricians)  Where the website has a search function, used search terms: “pregnancy blood pressure monitor” and “pregnancy blood pressure”. Additionally, sites were searched by clicking through links that were relevant to pregnancy and health. | **ONLINE**  Department of Health and Social Care  NHS Choices  NICE  Patientinfo.com  Royal College of Obstetrics and Gynaecology (RCOG)  Action on Pre-eclampsia (APEC)  Babycentre  Emma’s diary  Bounty  Boots |
| Email alerts | PPI women | **ONLINE**  Boots  Emma’s diary  NCT |
| Books | Searched for “pregnancy books” in  Google (www.google.co.uk)  Amazon (www.amazon.co.uk)  Waterstones (www.waterstones.com)  WH Smith books (www.whsmith.co.uk/books)  Books allocated “points” according to their position in each search where top book scores 10 points and 10^th^ scores 1 point. Scores for each book were added across the searches and top five scoring books selected.  Added “Your new Pregnancy Bible” as result of PPI | **OFFLINE**  Heidi Murkoff, *What to expect when you’re expecting*  Maggie Blott, *Day-to-Day Pregnancy Book*  Lesley Regan, *Your Pregnancy Week by Week*  Lisa Geddes, *Bumpology*  Fogle & Hunt, *The Bump Class*  Anne Deans, *Your New Pregnancy Bible The Experts' Guide to Pregnancy and Early Parenthood* |
| Magazines | Search magazine shelves in WH Smith  Hospital waiting area | **OFFLINE**  Mother & Baby  Bumps, birth and beyond  Emma’s diary |
| Apps (mobile software applications designed for smartphones) | Search “pregnancy” in Google Play app store and “pregnancy app” in search engine (Google).  Google search retrieves lists of apps recommended by: Netmums, Which, Healthline, Mother and Baby.  Apply inclusion/exclusion criteria. Identify 5 most commonly appearing apps in app stores and lists | **ONLINE**  What to expect pregnancy tracker  Baby bump  I’m expecting  Pregnancy +  Sprout |
| Discussion forums (user-generated health-related content) | Researchers, PPI group, HCPs | **ONLINE**  Mumsnet  Netmums  NCT  Babycentre  Mummy Pages  Health Unlocked  Neighbourhood midwives |
| General social media | Researchers, PPI group, HCPs | Facebook  Twitter |
| Blogs/Vlogs | Searched Google for “pregnancy health blogs”  [Initial, more complex search strategy not successful – see Appendix 3] | Healthline - top 10  Nutrition for health pregnancy - Wellness Mama  Pregnancy 1 – a healthy slice of life  Fit to be pregnant  Fit Pregnancy and baby  Fittamamma |
| Youtube | Search “Pregnancy high blood pressure” in Youtube | I am 25 weeks pregnant with high blood pressure. How can I lower it  Pregnancy update weeks 30-32 \| swelling and high blood pressure!  Pregnancy Tips : How to Reduce High Blood Pressure During Pregnancy  High blood pressure during pregnancy  High Blood Pressure during Pregnancy; Dr. Rathna Srinivasan, Fortis Healthcare India |
| Podcasts | Search Google “pregnancy podcast UK”  [only one potentially relevant podcast found] | Pea in the podcast |
